# Nitric Oxide Synthetic Pathway in Red Blood Cells Is Impaired in Coronary Artery Disease

**DOI:** 10.1371/journal.pone.0066945

**Published:** 2013-08-05

**Authors:** Sonia Eligini, Benedetta Porro, Alessandro Lualdi, Isabella Squellerio, Fabrizio Veglia, Elisa Chiorino, Mauro Crisci, Anna Garlaschè, Marta Giovannardi, Josè-Pablo Werba, Elena Tremoli, Viviana Cavalca

**Affiliations:** 1 Centro Cardiologico Monzino, Istituto di Ricovero e Cura a Carattere Scientifico (I.R.C.C.S.), Milan, Italy; 2 Dipartimento di Scienze Cliniche e di Comunità, Università degli Studi di Milano, Milan, Italy; 3 Dipartimento di Scienze Farmacologiche e Biomolecolari, Università degli Studi di Milano, Milan, Italy; Universidade de São Paulo, Brazil

## Abstract

**Background:**

All the enzymatic factors/cofactors involved in nitric oxide (NO) metabolism have been recently found in red blood cells. Increased oxidative stress impairs NO bioavailability and has been described in plasma of coronary artery disease (CAD) patients. The aim of the study was to highlight a potential dysfunction of the metabolic profile of NO in red blood cells and in plasma from CAD patients compared with healthy controls.

**Methods:**

We determined L-arginine/NO pathway by liquid-chromatography tandem mass spectrometry and high performance liquid chromatography methods. The ratio of oxidized and reduced forms of glutathione, as index of oxidative stress, was measured by liquid-chromatography tandem mass spectrometry method. NO synthase expression and activity were evaluated by immunofluorescence staining and *ex-vivo* experiments of L-[^15^N_2_]arginine conversion to L-[^15^N]citrulline respectively.

**Results:**

Increased amounts of asymmetric and symmetric dimethylarginines were found both in red blood cells and in plasma of CAD patients in respect to controls. Interestingly NO synthase expression and activity were reduced in CAD red blood cells. In contrast, oxidized/reduced glutathione ratio was increased in CAD and was associated to arginase activity.

**Conclusion:**

Our study analyzed for the first time the whole metabolic pathway of L-arginine/NO, both in red blood cells and in plasma, highlighting an impairment of NO pathway in erythrocytes from CAD patients, associated with decreased NO synthase expression/activity and increased oxidative stress.

## Introduction

Nitric oxide (NO) is a signaling molecule that has a pivotal role in regulating vascular tone. It promotes several beneficial effects in the vasculature, favoring vasodilatation and inhibiting smooth muscle cells proliferation, enhancing fibrinolysis, and inhibiting some activities of circulating blood cells, as platelet aggregation and leukocyte adhesion [1], [2]. NO is synthesized by a family of NO synthases (NOSs) through the conversion of L-arginine (Arg) to L-citrulline (Cit). Endogenous Arg analogues, the dimethylarginines (DMAs), are able to inhibit NO synthesis. In particular, asymmetric dimethylarginine (ADMA) competes with the substrate at the catalytic site of NOS and symmetric dimethylarginine (SDMA) interacts with the transport of Arg into the cells, via the transporter for cationic amino acids (CAT). Increased plasma levels of these DMAs have been described in coronary artery disease (CAD) [3].

Endothelial cells are the main producers of NO, but other circulating cells are involved in NO synthesis, i.e. platelets, monocytes and red blood cells (RBCs). Initially, it has been observed that RBCs are able to scavenge NO synthesized by endothelial cells, providing the transport of oxidized (nitrite/nitrate) and nitrosylated (SNO-Hb and HbNO) forms of NO in the bloodstream and their local delivery [4]. More recently, it has been shown that RBCs are able to synthesize NO through a constitutive type of NOS (RBC-NOS), which is similar to the enzyme found in endothelial cells [5].

All the enzymes involved in DMAs metabolism (synthesis or catabolism) [6] as well as the CAT have been found in RBCs [7]. In addition, large amounts of ADMA and SDMA have been evidenced into RBC proteins [8], [9].

Some authors have investigated the role of RBC-derived NO in the regulation of blood flow [10] and platelet function [5], [11]. Even if, up to now, no clinical implications of the alteration of this NO source have been depicted, a stimulation or an inhibition of RBC-NOS results in a decrease or an increase of platelet aggregation, respectively [5]. RBC-derived NO also acts in an autocrine manner by modulating the deformability of RBCs thus favoring their passage through the capillaries and improving the blood flow in the microcirculation [12], [13]. Recently, RBC-NOS activity has been reported to be impaired in CAD patients [14].

Endothelial dysfunction, with reduced NO bioavailability, is a pathological condition frequently occurring in CAD patients [15]. An increased oxidative stress may reduce the NO bioavailability through an impairment of the NO synthesis and through the inactivation of the NO produced by transforming it into peroxynitrate. Oxidative stress, resulting from the imbalance between oxidant factors and antioxidant defense systems, has been previously reported in CAD patients [16], [17].

In this study, we hypothesized that reduction of NO biosynthesis occurs in CAD RBCs and that it may be ascribed to a dysregulated Arg metabolism and/or increased oxidative stress. To this aim we investigated the synthetic and metabolic profile of NO and oxidative stress both in RBCs and in plasma from healthy subjects and from patients affected by CAD.

## Methods

### Ethical approval

This observational study was conducted with the approval of the local ethics research committee of Centro Cardiologico Monzino (n° S1687/610) and written informed consent to participate was obtained from all subjects. The investigation conformed to the principles outlined in the Declaration of Helsinki.

### Study population

Patients with stable effort angina or inducible ischaemia and documented CAD were enrolled. Eligibility of patients was based on the presence of stable exertional angina and positive stress test, as judged by at least 1.5 mm horizontal or down-sloping ST-segment depression. Key angiographic inclusion criteria was the evidence of >75% narrowing in at least one major coronary vessel, with normal left ventricular ejection fraction (≥50%) assessed by two-dimensional echocardiography. Patients with a history of congestive heart failure, significant valvular diseases, hypertrophic cardiomyopathy, vasospastic angina, recent (<6 months) acute coronary syndrome, surgical or percutaneous revascularization, and those with pacemaker dependency and atrial fibrillation were excluded. Patients with renal insufficiency (serum creatinine concentration >1.4 mg/dL), hepatic disease, recent infection, recent major surgical interventions, immunological disorders, chronic inflammatory or neoplastic diseases, were also excluded. Twenty-two patients met the eligibility criteria.

Twenty healthy subjects without cardiovascular risk factors and evidence of CAD were enrolled as control group (Ctrl) from those attending the clinic for global control of cardiovascular risk at Centro Cardiologico Monzino IRCCS.

### Blood collection

Peripheral blood was drawn from the antecubital vein of patients and controls while fasting, into tubes containing EDTA (9.3 mM; Vacutainer Systems, Becton Dickinson, Franklin Lakes, NJ, USA) to obtain whole blood, plasma and erythrocyte samples. EDTA-anticoagulated blood was centrifuged at 1.200 *g* for 10 min at 4°C. Plasma was separated and aliquots were stored at −80°C until analyses. Aliquots of packed red cells were lysed by cold deionized water to obtain lysed RBCs and stored at −80°C until analyses.

### Biochemical determinations

#### L-arginine/NO metabolome

Arg/NO pathway was determined both in lysed RBCs and in plasma. Arg, ADMA, SDMA, Cit and L-ornithine (Orn) were simultaneously measured by liquid chromatography – tandem mass spectrometry (LC-MS/MS) as previously described [18].

The ratio Arg/(Orn+Cit) as index of global Arg availability [19], [20] and the ratio Orn/Cit as indicator of the relative activity of arginase and NOS [21] were computed.

#### Tetrahydrobiopterin

Tetrahydrobiopterin (BH_4_) concentration was measured in lysed RBCs and in plasma by HPLC after selective oxidation with iodine, using the method described by Fukushima and Nixon [22].

#### Glutathione

Reduced glutathione (GSH) and disulphide glutathione (GSSG) were measured on whole blood added with 10% trichloroacetic acid in 1 mM EDTA solution to precipitate proteins and stored at −80°C until analysis. Levels of GSH and GSSG were assessed by LC-MS/MS method [23]. Levels of GSH and GSSG were expressed as µmol/g Hb.

### RBC-NO synthase expression

#### Immunofluorescence and confocal microscopy

The confocal analysis was performed in a subgroup of subjects (n = 10 per group matched for age and sex, randomly chosen and representative of the enrolled population). After plasma separation, an aliquot of RBCs was fixed in 2% paraformaldehyde at room temperature (RT) for 30 min, stroked on glass and heat fixed. Non specific reactive sites were blocked with 5% bovine serum albumin solution containing 0.1% saponin for 30 min at RT. RBCs were incubated overnight at 4°C with a monoclonal anti eNOS antibody (2.5 µg/ml; BD Biosciences, Milano, Italy) followed by washings and incubation with an anti-mouse conjugated secondary antibody (40 µg/ml; Alexa Fluor488) for 1 hr at RT. The samples were mounted with fluorescent mounting medium (DAKO, Milano, Italy) and examined by laser scanning confocal microscope (LSM710, Carl Zeiss, Milano, Italy) using a 63×/1.3 oil immersion objective lens. For negative controls, RBCs were incubated in the absence of primary antibody. Fluorescent images were captured on confocal microscope connected to a digital camera using the image processor Zen (Carl Zeiss). For analysis of immunostaining intensity in RBCs, the grey values of Ctrl and CAD patients from at least three randomly selected areas of each smear, were measured. The fluorescence immunostaining is reported as the mean of grey value subtracted of background grey value determined on the same smear in the absence of primary antibody.

### In vitro RBC-NO synthase activity

NOS activity in RBCs was measured in a subgroup of age and sex matched subjects (n = 8 Ctrl and CAD patients, randomly chosen and representative of the enrolled population). The enzymatic activity was measured *in vitro* by the conversion of L-[^15^N_2_]arginine to L-[^15^N]citrulline in the presence of the arginase inhibitor N(omega)-hydroxy-nor-l-arginine (nor-NOHA). Washed RBCs (10^6^cells/µl) were lysed on ice by cold deionized water (1∶1, v/v) in the presence of protease inhibitors (phenylmethanesulfonylfluoride, 2 mM; leupeptin, 4 µM; aprotinin, 4 µM). Samples were incubated at 37°C for 2 hr with L-[^15^N_2_]arginine (75 µM) and NOS cofactors in the absence or in the presence of nor-NOHA (50 µM). The composition of reaction buffer (in µM) was as follows: Tris-HCl, 250, pH 7.4; CaCl_2_, 500; BH_4_, 0.3; flavin adenine dinucleotide (FAD), 0.1; flavin mononucleotide (FMN), 0.1; nicotinamide adenine dinucleotide phosphate (NADPH), 100. The reaction was stopped by the addition of 5 volumes of acetonitrile/methanol (50∶50, v/v). Precipitated proteins were separated by centrifugation at 10.000 *g* for 15 min at 4°C and stored at −80°C until analysis. The analytes were measured by LC-MS/MS and the activity was quantified as the ratio between the L-[^15^N]citrulline (µmol/10^6^ cells) and residual L-[^15^N_2_]arginine (mmol/10^6^ cells) [21].

### Statistical analysis

Twenty subjects per group allowed a statistical power of 90% to deem as significant a between-group difference in any analyte approximately equal to one standard deviation, with an alpha error of 0.05. Variables with skewed distribution were log transformed before analysis. Patient's characteristics were compared by T-test. Oxidative stress and Arg/NO pathway variables were compared between CAD and Ctrl by covariance analysis, adjusting for age and sex. Immunofluorescence intensity was compared between groups by repeated measures covariance analysis, taking into account triplicate measures for each subject. Unadjusted associations between analyte concentrations were assessed by bi-variate Pearson correlation; partial correlations were also computed in order to adjust for the effects of the other analytes. All analyses were performed by SAS v. 9.2 (SAS Institute Inc., Cary, NC, USA).

## Results

### Population

Demographic and clinical characteristics of CAD patients and controls are reported in Table 1. CAD patients were older and had higher BMI. There were more hypercholesterolemic and hypertensive subjects among CAD patients, but both LDL cholesterol and systolic/diastolic pressure values were similar between patients and Ctrl, as the result of pharmacological treatments.

**Table 1:** Demographic and clinical characteristics of CAD patients and Ctrl subjects.

Variable	CAD (n = 22)	Ctrl (n = 20)	*P value*
Age (years)	66.1±8.6	55.5±10.2	0.002
Male gender	17(77.3)	14(70)	0.592
BMI	27.3±3.11	24.36±2.35	0.005
Total cholesterol (mg/dL)	208.3±29.2	208.1±26.8	0.95
HDL-cholesterol (mg/dL)	46.9±17.2	54.5±17.3	0.2
LDL-cholesterol (mg/dL)	133.8±36.8	132.3±22.1	0.91
Triglycerides (mg/dL)	131.5±69.4	95.5±32.5	0.11
Systolic Blood pressure (mmHg)	138.9±18.3	130±14	0.08
Diastolic Blood pressure (mmHg)	80.0±9.1	78±6	0.4
Creatinine (mg/dL)	0.86±0.22	0.81±0.14	0.46
Current Smoker	3(13.64)	0(0)	0.12
HyperCholestolemia	14(63.6)	2(10.0)	0.0004
IperTrygliceridemia	2(9.1)	1(5.0)	0.75
Hypertension	14(63.6)	2(10.0)	0.0004
Pharmacological treatments			
Converting enzyme inhibitors	6(27.3)	0(0)	
Antiplatelets	4(18.2)	0(0)	
Aspirin	13(59.1)	0(0)	
Beta-Blockers	5(22.7)	1(5.0)	
Calcium channel blockers	4(18.2)	1(5.0)	
Diuretics	2(9.1)	0(0)	
Statins	14(63.6)	2(10.0)	
Hypoglycemics	0(0)	0(0)	
Angiotensin receptor blockers	6(27.3)	0(0)	

Quantitative variables were expressed as mean ± SD and categorical variables as n (%).

P value: Wilcoxon test for continous variables and Chi Square test for categorical variables.

P value adjusted for sex and age after log-transformation of the data.

### Assessment of products of the Arg/NO pathway in plasma and RBCs

**Figure 1:**
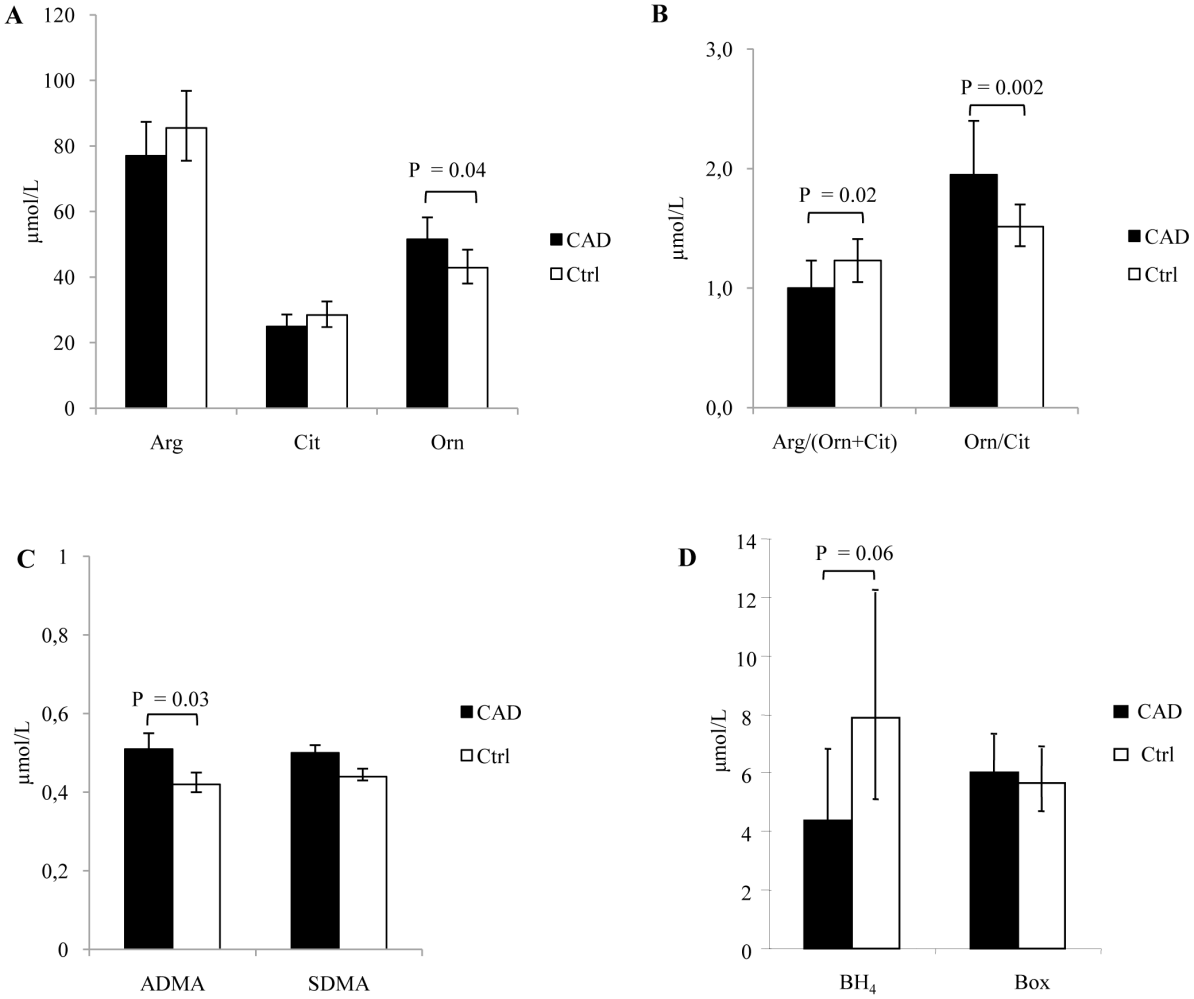
Plasma levels of analytes involved in Arginine/NO pathway. (A) L-arginine (Arg), L-citrulline (Cit), L-ornithine (Orn) were simultaneously determined by LC-MS/MS. (B) Arg bioavailability and the relative activity of arginase and NOS enzymes are expressed as Arg/(Orn+Cit) and Orn/Cit ratios, respectively. (C) The endogenous inhibitors asymmetric dimethylarginine (ADMA) and symmetric dimethylarginine (SDMA) were determined by LC-MS/MS. (D) Tetrahydrobiopterin (BH_4_) and oxidized biopterins (Box) were detected by HPLC after selective oxidation with iodine. Data are presented as age and sex adjusted geometric means and 95% C.I. Comparisons between groups (CAD, n = 22; Ctrl, n = 20) were performed by covariance analysis, adjusting for age and sex.

**Figure 2:**
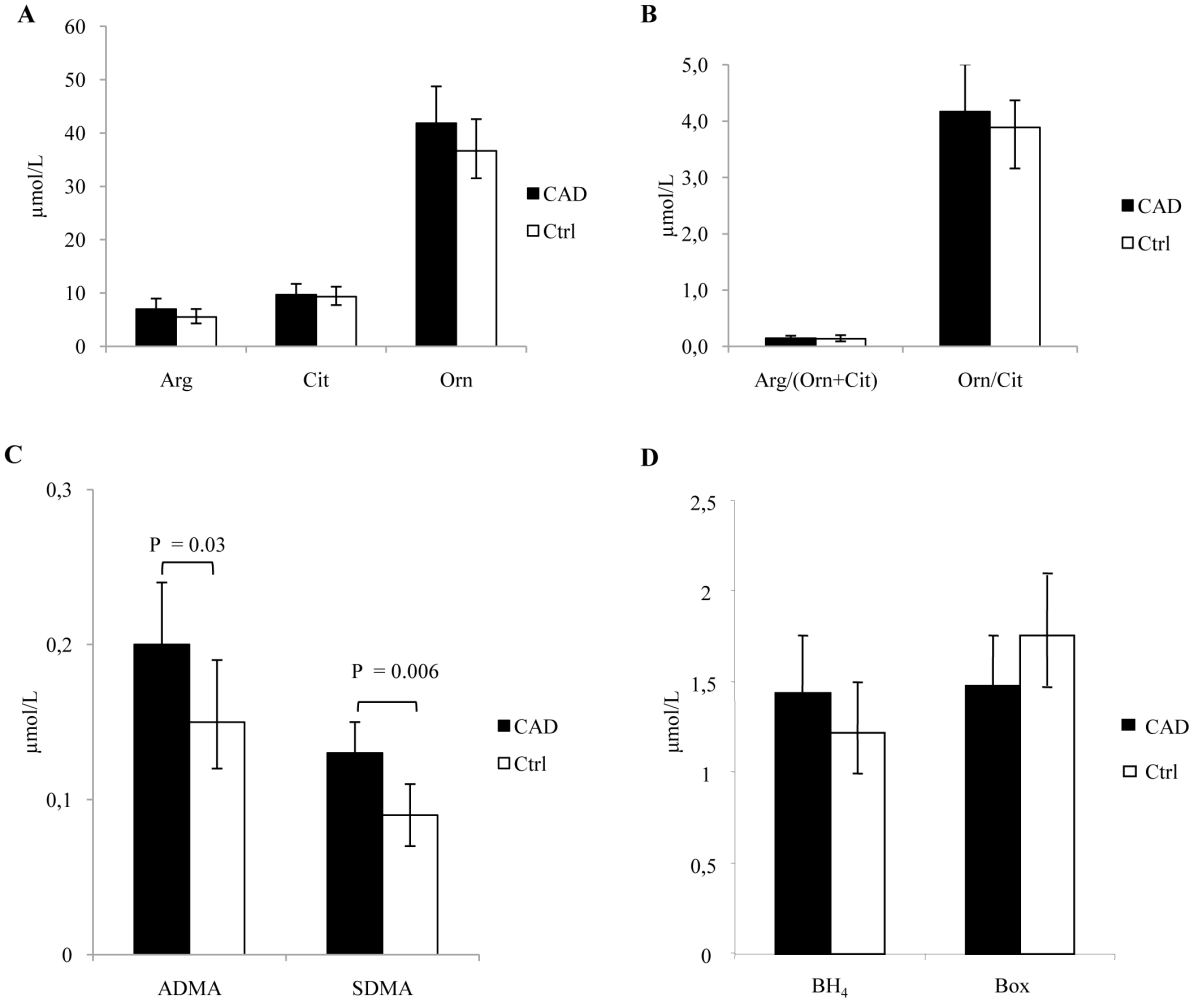
RBC levels of analytes involved in Arginine/NO pathway. (A) L-arginine (Arg), L-citrulline (Cit), L-ornithine (Orn) were simultaneously determined by LC-MS/MS. (B) Arg bioavailability and the relative activity of arginase and RBC-NOS enzymes are expressed as Arg/(Orn+Cit) and Orn/Cit ratios, respectively. (C) The endogenous inhibitors asymmetric dimethylarginine (ADMA) and symmetric dimethylarginine (SDMA) were determined by LC-MS/MS. (D) Tetrahydrobiopterin (BH_4_) and oxidized biopterins (Box) were detected by HPLC after selective oxidation with iodine. Data are presented as age and sex adjusted geometric means and 95% C.I. Comparisons between groups (CAD, n = 22; Ctrl, n = 20) were performed by covariance analysis, adjusting for age and sex.

In the study we simultaneously measured Arg, Cit, Orn and the endogenous inhibitors ADMA and SDMA in plasma and in lysed RBCs from CAD patients and Ctrl by LC-MS/MS (Figure 1 and 2). In plasma (Figure 1A) mean levels of Arg and Cit were comparable between the two groups, whereas levels of Orn were higher in CAD patients than in Ctrl. As a consequence, the ratio Arg/(Orn + Cit), as index of Arg bioavailability, was lower in CAD than in Ctrl (Figure 1B). In addition the Orn/Cit ratio in plasma differed significantly in the two groups (Figure 1B), suggesting different activities of the Arg metabolic enzymes, i.e. arginase and eNOS. Furthermore, mean levels of ADMA and SDMA (Figure 1C) were higher in CAD patients than in Ctrl, whereas the levels of cofactor BH_4_ (Figure 1D) were lower, the difference approaching statistical significance.

In RBCs the levels of Arg, Orn and Cit (Figure 2A), and of the cofactor BH_4_ (Figure 2D), were comparable between the two groups. Consequently the Arg bioavailability and Orn/Cit ratio were similar in the two groups (Figure 2B).

Mean levels of ADMA and SDMA, instead, were significantly greater in RBCs of CAD patients compared to Ctrl (Figure 2C).

Tables 2 and 3 show the correlation pattern between plasma and RBC analytes involved in NO biosynthesis in CAD patients and in Ctrl. Even if many of the analytes were significantly associated in both groups (Table 2), after mutual adjustment several correlations disappeared (Table 3). Interestingly, a significant and independent correlation between Cit levels in RBCs and in plasma was found in both groups.

**Table 2:** Unadjusted simple Pearson correlation pattern between plasma and RBC analytes.

CAD						Ctrl					
Plasma arginine						Plasma arginine					
0.33	**Plasma ornithine**					0.49	**Plasma ornitine**				
**0.74** ‡	0.38	**Plasma citrulline**				**0.74** ‡	**0.54** †	**Plasma citrulline**			
**0.70** ‡	0.00	**0.56** †	**RBC arginine**			0.18	0.11	0.04	**RBC arginine**		
0.36	**0.46** *	0.34	0.15	**RBC ornithhine**		0.28	**0.48** *	**0.68** †	0.08	**RBC ornithine**	
**0.59** †	0.31	**0.68** ‡	**0.44** *	**0.66** ‡	**RBC citrulline**	0.33	0.26	**0.75** ‡	0.13	**0.89** ‡	**RBC citrulline**

* P<0.05,

† P<0.01,

‡ P<0.001.

**Table 3:** Partial Pearson correlation pattern between plasma and RBC analytes, adjusted for the effects of all other analytes.

CAD						Ctrl					
Plasma arginine						Plasma arginine					
	**Plasma ornithine**						**Plasma ornithine**				
**0.69** †		**Plasma citrulline**				**0.72** ‡		**Plasma citrulline**			
**0.56** *			**RBC arginine**						**RBC arginine**		
				**RBC ornithine**			**0.66** †			**RBC ornithine**	
		**0.50** *		**0.63** †	**RBC citrulline**			**0.65** †		**0.84** ‡	**RBC citrulline**

* P<0.05,

† P<0.01,

‡ P<0.001.

### RBC-NO synthase expression

**Figure 3:**
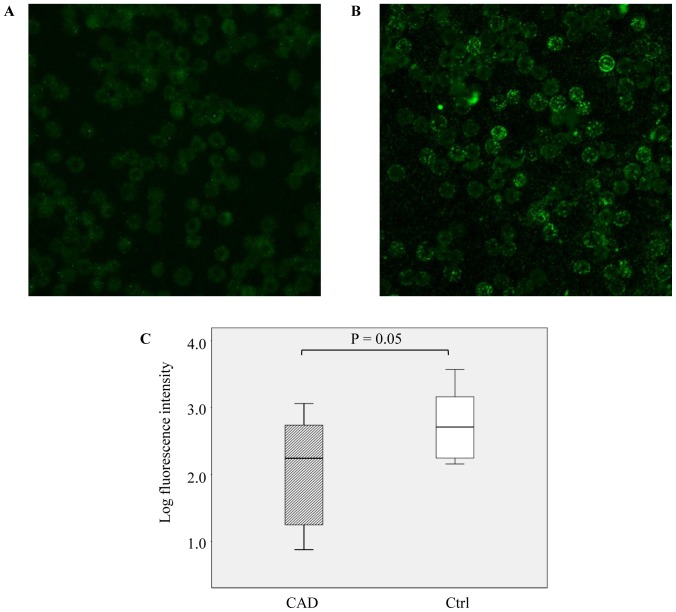
Immunostaining of NO synthase (NOS) protein in human red blood cells (RBCs). NOS was detected in RBCs from CAD patients (A) and from Ctrl (B). RBCs were incubated with a monoclonal anti eNOS antibody (2.5 µg/ml) and with an anti-mouse conjugated secondary antibody (40 µg/ml; Alexa Fluor488). The samples were mounted with fluorescent mounting medium and examined by laser scanning confocal microscope (LSM710, Carl Zeiss) using a 63×/1.3 oil immersion objective lens. Fluorescent images were captured with a digital camera using the image processor Zen (Carl Zeiss). (C) Fluorescence intensity (densitometric sum of grey) was quantified. Data are expressed as the log median of total fluorescence intensity per field ± interquartile range subtracted of the negative control value. Means derive from n = 10 CAD and n = 10 Ctrl.

Confocal microscopy of washed RBCs revealed a distinct ring of immunofluorescence staining surrounding the cytoplasm of RBCs and, to a lesser extent, punctuate immunofluorescence structure through the entire cytoplasm (Figure 3A and B from CAD patients and Ctrl, respectively). Median fluorescence intensity was significantly lower in CAD patients than in Ctrl (Figure 3C).

### RBC-NO synthase activity

The activity of RBC-NOS *in vitro* was tested by measuring the hydrolysis of the L-[^15^N_2_]arginine to the labeled product L-[^15^N]citrulline, which during the reaction is produced in equimolar amounts as NO. After incubation of RBCs with L-[^15^N_2_]arginine a marked accumulation of Orn was found (about 160% increase in both groups, corresponding to a mean increment over basal of 26.3±8.3 and of 29.1±9.8 µmol/10^6^ cells in CAD patients and Ctrl, respectively, p = 0.001 for both). These data documented the predominant activity of arginase *vs* NOS in RBCs.

**Figure 4:**
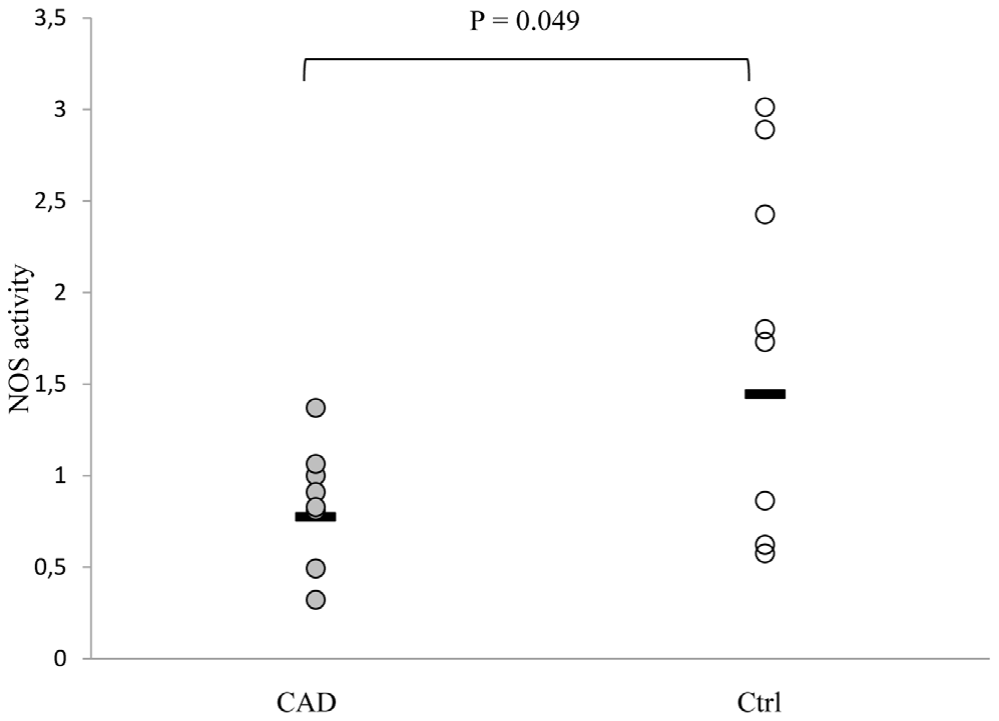
NO synthase (NOS) activity in lysed human red blood cells (RBCs). RBCs were washed and lysed with H_2_O containing protease inhibitors. RBC-NOS activity was measured after the addition of L-[^15^N_2_]arginine in the presence of arginase inhibitor nor-NOHA (50 µM). The levels of L-[^15^N]citrulline and L-[^15^N_2_]arginine were determined by LC-MS/MS. The RBC-NOS activity was evaluated by the ratio between L-[^15^N]citrulline formed and L-[^15^N_2_]arginine residue. Individual data are represented as geometric means for CAD (n = 8) and Ctrl (n = 8).

In the presence of arginase inhibitor Nor-NOHA the RBC-NOS activity, assessed by the ratio between the L-[^15^N]citrulline and residual L-[^15^N_2_]arginine, was significantly reduced in CAD with respect to Ctrl (ratio geometric mean and 95% confidence interval: 0.78, 0.54–1.15 *vs*. 1.45, 0.83–2.57 for CAD and Ctrl respectively, P = 0.049), (Figure 4).

### Oxidative stress in RBCs

Whole blood of CAD patients had lower GSH levels than that of Ctrl (5.97±0.3 and 7.70±0.3 µM/g Hb, mean±SE, respectively, P<0.0001) and higher levels of GSSG (0.62±0.04 and 0.29±0.04 µM/g Hb, mean±SE, respectively, P<0.0001). The GSSG/GSH ratio, recognized index of oxidative stress [24], [25], was accordingly greater in CAD patients than in Ctrl (0.11±0.01 and 0.04±0.001 respectively, P<0.0001), which documents a higher level of oxidative stress in RBCs of CAD patients. Finally, a positive correlation between the GSSG/GSH and Orn/Cit ratios (r = 0.56, P = 0.007) was found in RBCs of CAD patients.

## Discussion

Alterations of RBC compartment associates with a poor prognosis in patients with coronary disease and plasma hemoglobin is an independent predictor of major adverse cardiovascular events in patients with acute coronary syndromes [26], [27]. Initially, it was hypothesized that RBCs serve as scavengers or transporters for NO produced by endothelial cells [28] but, recently, an active role of RBCs in NO biosynthesis has been recognized [5].

In this study we have investigated the overall metabolic pathway involved in NO biosynthesis in RBCs and we have measured the levels of products involved in this pathway in plasma. We report here unprecedented data that depict an impairment of NO biosynthetic pathway in RBCs of CAD patients, compared to those of healthy subjects. This impairment might be partially ascribed to the higher levels of ADMA and SDMA in RBCs from CAD compared to Ctrl. Increased levels of ADMA and SDMA in plasma of patients with cardiovascular disease have been previously reported by others and by our group [29]–[31]. The impaired renal function of CAD patients has been considered to be the main reason for these findings [3], [32]. Patients in this study, however, had a renal function within the normal range, and, accordingly, plasma levels of SDMA were similar to Ctrl. Instead, RBC levels of SDMA in these patients were significantly greater than in Ctrl.

Concerning the other factors involved in NO biosynthesis, several observations were done. First, Arg bioavailability, e.g. Arg/Orn+Cit, and the Orn/Cit ratio were comparable between patients and Ctrl in RBCs, but not in plasma. This finding points out toward a potential dynamic equilibrium, which is relevant in order to maintain intracellular homeostasis. The higher levels of Orn in plasma of CAD patients reflect the preponderance of the metabolic pathway that transforms Arg into Orn.

Finally, Cit levels were similar in patients and in controls both in plasma and in RBCs. In this context it is of relevance to underline that Cit may derive not only from the activity of NOS on Arg, but also from that of dimethyl arginine dimethylaminohydrolase (DDAH) on ADMA. It should be noted, however, that these pathways supply only a minor fraction of plasma Cit as its major source is the small intestine [33]. Indeed, in our opinion, the lack of difference in Cit levels between patients and Ctrl is due to a continuous exchange between the cellular and plasma compartments, rather than to a direct effect of the degradation of ADMA, as the presence of DDAH in RBCs is controversial [9], [34]. Indeed, a positive correlation between the plasmatic level and the RBC content of Cit was observed.

The assessment of the NO pathway in RBCs has to take into account the expression and activity of the NO synthase enzyme. In agreement with previous studies, we detected the presence of NOS in RBCs mainly in the membrane compartment, and this compartmentalization might be relevant for the export of NO toward other cells or to plasma, as previously suggested by Cortese-Krott et al [14]. In addition, intracellular NO in RBCs could induce electromechanical modifications of proteins and lipids/lipoproteins present in the membranes, thus preventing NO consumption by RBCs [35]. Although the presence of NOS in RBCs has been confirmed, data concerning the activity of this enzyme are still controversial [5], [11], [14], [36], [37]. However variable experimental conditions and methodological shortcomings were reported. For instance, Kang et al [37] carried out the study in homogenized cell fractions and it is possible that cofactors necessary for NOS activity such as FAD and BH_4_ are lost during homogenization as suggested by Mehta [38]. Bohmer [36] did not find a functional RBC-NOS but he didn't take into consideration the activity of arginase.

In our study NOS activity in RBCs was measured in cell lysates, in order to preserve the whole metabolic cellular system, by the formation of L-[^15^N]citrulline *in vitro*, after the inhibition of arginase. Indeed, arginase, by competing with NOS for the substrate Arg, can limit its availability for NO biosynthesis in intact cells. Although the affinity of Arg is much higher for purified NOS (Km approximately 2–20 µM) than for arginase (Km approximately 2–20 mM), the maximum activity of arginase at physiological pH is 1000-fold greater than that of NOS [39]. Indeed, overexpression of the two isoforms of arginase (isoforms 1 and 2) in endothelial cells has been reported to suppress NO generation [40], which suggests that the inhibition of the two arginase isoforms is associated with an increase of NO biosynthesis by endothelial cells [41]. In addition, in coronary arteries of diabetic rats, an increased arginase activity resulted in a reduced bioavailability of Arg as a substrate for eNOS [42]. The importance of endothelial arginase in different pathological conditions as hypertension, diabetes , ischaemia-reperfusion, cystic fibrosis, sickle cell disease and asthma has already been reported [43]. Indeed, in RBCs of hypertensive patients, increased arginase activity accompanied by reduced levels of nitrite and nitrates was found [44].

Overall, increased arginase activity in endothelial cells has been proposed to promote a proatherogenic effect due to the reduction of cell NO biosynthesis [45]. Accordingly, our data show that in RBCs the substrate Arg is preferentially metabolized by the arginase enzyme as documented by the selective increase in Orn levels after the addition to RBCs of L-[^15^N_2_]arginine. This predominant activity of arginase might explain the apparent inconsistency of our data with those previously published by others, which reported that NOS enzyme in RBCs is inactive [36], [37].

Interestingly, we found that the lower expression of NOS detected by confocal microscopy in RBCs of CAD patients went in parallel with a reduced activity of the enzyme. We cannot exclude the effects of pharmacological treatments, ongoing in the patients, on NOS activity that can be the result of the reduced expression of the enzyme and/or of the increased levels of ADMA and SDMA. Indeed some authors evidenced that an angiotensin converting enzyme inhibitor, lisinopril, monotheraphy or combined with statin therapy, decreases arginase activity in RBCs [44]. In addition statins [46] and aspirin [47] were described to induce NOS activity in RBC *in vivo*. On these bases, these drugs could result in an increase of NO RBC production masking a more conspicuous difference in CAD *vs* Ctrl.

Arg metabolic pathway may be impaired by the occurrence of oxidative stress, which in turn stimulates arginase activity [43] and conversely inhibits NOS activity [48], [49]. Indeed, in agreement with previous studies [17], [50], [51], we found reduced levels of GSH in CAD patients. The positive correlation between GSSG/GSH and Orn/Cit found in our patients further supports the predominant activation of arginase over NOS activation in the presence of oxidative stress.

In conclusion our study shows for the first time that RBCs of CAD patients have an impairment of the NO pathway, decreased NOS expression/activity and increased oxidative stress. Therapeutic interventions aimed at reducing intracellular oxidative stress, e.g. restoration of GSH levels, might be effective in improving the balance between NOS and arginase activities.
